# Central adiposity as a predictor of mortality in older adults: Identification of cutoffs using generalized additive models

**DOI:** 10.3389/fnut.2023.1132006

**Published:** 2023-04-18

**Authors:** Dalila Pinto de Souza Fernandes, Leidjaira Lopes Juvanhol, Aline Araújo Nobre, Ângela Maria Natal de Souza, Andréia Queiroz Ribeiro

**Affiliations:** ^1^Department of Nutrition and Health, Federal University of Viçosa, Viçosa, Brazil; ^2^Scientific Computing Program, Oswaldo Cruz Foundation, Rio de Janeiro, Brazil; ^3^Department of Population Studies, Institute Abel Salazar, University of Porto, Porto, Portugal

**Keywords:** anthropometry, central adiposity, cutoff points, mortality, older adults

## Abstract

**Background:**

Obesity is associated with premature mortality in adults; however, this association has been inconsistent in the older adult population. In addition, there is a lack of specific cutoff points for indicators of negative health outcomes in older adults. Methods: This is a prospective study with 796 non-institutionalized older adults. Data on sociodemographic characteristics, lifestyle, food consumption, and nutritional status were obtained at baseline. Generalized additive models were used to identify cutoff points for the waist circumference (WC) and waist-to-height ratio (WHtR) and Cox proportional hazards models to assess the independent association between adiposity and mortality.

**Results:**

Over the 9 years of follow-up, 197 deaths (24.7%) occurred, of which 51.8% were men, with a mean age of 76.1 ± 9.0 years. Older adults at higher risk of death had WHtR of <0.52 or ≥0.63 and WC of <83 cm or ≥101 cm. An increased risk of death was observed in older adults with high WC (HR: 2.03 95% CI: 1.20–3.41) and high WHtR (HR: 1.51 95% CI: 1.01–2.26) in the adjusted models, and an increase in WC was a risk factor for higher CVD mortality (HR: 2.09, 95% CI: 1.12–3.88) in the adjusted models.

**Conclusion:**

Adiposity was associated with an increased risk of death in older adults. In view of these results and considering the lack of cutoff points for anthropometric indices in Brazilian older adults, further studies are needed to confirm the WC and WHtR cutoff values found in this study.

## Introduction

Obesity, an important risk factor for chronic diseases, is associated with premature mortality in adults (1); however, this association has been inconsistent in the older population (2, 3). Several studies reported a decrease in the mortality rate in older adults who were overweight or obese compared to those with normal weight (4, 5), and this protective effect of excess weight on survival is called the “obesity paradox” (6). Other studies did not find this to be the case (7, 8). Nevertheless, most studies that support the obesity paradox used the body mass index (BMI) to measure excess weight.

Although BMI is widely used in epidemiological studies and incorporated into clinical practice due to its simplicity in application, it cannot distinguish between lean and fat mass (9, 10). In addition, studies suggest that the information produced by the BMI is associated with other measures of adiposity, such as waist circumference (WC), waist-hip ratio (WHR), and waist-to-height ratio (WHtR), which are considered the best predictors of abdominal adiposity and have been associated with higher mortality in older adults (11, 12), including mortality by cardiovascular diseases (CVDs) (13). Another issue, in this context, is the absence of specific cutoff points for these indicators to predict negative health outcomes in older adults. Furthermore, both low weight and adiposity have been associated with an increased risk of death.

In view of the inconsistency of the results and the scarcity of studies about the relation between WC and WHtR and mortality in older adults, this study aimed to investigate the association between central adiposity and all-cause mortality and also CVD mortality in the older population and estimate cutoff points for the anthropometric indices of WC and WHtR.

## Methods

### Study design and sample plan

This is a prospective study conducted in a medium-sized city in southeast Brazil (Viçosa). The sample was obtained from 7,980 non-institutionalized older adult residents in the city aged 60 or older.

The data used in this study originated from previous research conducted to assess the nutritional status and health conditions of older adults (14). The population was identified by census during the National Elderly Vaccination Campaign in 2008 (80% of vaccination coverage). This census generated a database, which was supplemented with information from the records of the active and retired employees of the Federal University of Viçosa, the Family Health Program, the Municipal Physiotherapy Service, the Women’s Health Center, the Psychosocial Service, the Hiperdia Program, and the Municipal Polyclinic. This databank was organized in alphabetical order for sampling purposes.

The sample size was calculated considering a 95% confidence level, a prevalence of 50% (due to the multiple outcomes of interest from the major project) (14), and a tolerated error of 3.5%. From these parameters, the minimum final sample was estimated at 714 individuals. To this amount, 20% was added to cover possible losses, totaling 858 older adults. The selection was made by simple random sampling. After excluding losses, 796 older adults constituted the final sample. Participation does not differ significantly according to sex. However, non-participants are older than participants (*p* < 0.05; Supplementary Table 1).

### Eligibility criteria

Inclusion criteria consisted of older adults with an interest in participating in the study and participants aged 60 years or older.

Non-inclusion criteria consisted of individuals whose address could not be found, had died, and those with physical limitations (e.g., wheelchair user, limb amputation, or being bedridden), which prevented anthropometric measurements.

### Data collection

Baseline data (June 2009–December 2010) were collected at home, using a structured questionnaire and anthropometric assessment. A portable scale with a capacity of 199.95 kg and accuracy of 50 g (LC 200 pp., Marte Balanças e Aparelhos de Precisão Ltda.®, Brazil) was used to measure weight. The participants wore light clothing, no shoes, and no jewelry. Height was measured with a stadiometer with a precision of 0.35 -2.13 m and of 0.1 mm precision (Alturaexata®, Brazil). Participants stood barefoot,with their heels together, in an upright position, leaning against the wall and gaze fixed at the height of the horizon (15). WC was measured using a flexible and inelastic measuring tape, with 1.80 m length and 0.1 mm precision, adjusted to the body, without compressing the underlying soft tissue. The subject remained in an orthostatic position, and WC was measured at the midpoint between the last rib and the iliac crest (16).

The second stage consisted of collecting information about the deaths that occurred during the study period (June 2009–July 2018) from the Brazilian Mortality Information System (SIM), the Municipal Health Department of Viçosa. The death records of the baseline participants were identified by their full names and their mothers and date of birth. These data were compared to the personal information in the questionnaire to avoid the occurrence of homonyms. After identification and comparison, the information regarding the underlying cause of death was extracted from the SIM.

If there were no records of the participant in the SIM, their families were contacted by telephone to confirm that they were alive. When the participants were identified as deceased, home visits to family members/guardians were scheduled to collect information on the date, place, and underlying cause of death from the death certificate. The researchers went directly to the addresses of the subjects to obtain information if there was no information about the telephone number at the baseline or after three unsuccessful attempts to contact the participant by telephone. Finally, where there was a lack of information on the occurrence or not of death and those who had moved outside the municipality without leaving a new address were considered losses (Supplementary Figure 1).

The research was conducted according to the Declaration of Helsinki, and all participants signed the informed consent form before the interview and assessments. This study was approved by the Human Research Ethics Committee of the Federal University of Viçosa/MG (CAAE: 65782817.1.0000.5153).

### Study variables

Mortality was considered the outcome variable.

The anthropometric indicators of adiposity WC and WHtR were considered as the exposure variables. The WHtR is the ratio between WC (cm) and height (cm).

To define the adjustment models, variables were initially selected from the literature review, and subsequently, a theoretical model was established. After that, the variables were confirmed as confounding factors or potentially mediating by the directed acyclic graph (DAG) tool in the DAGitty program^1^ (Supplementary Figure 2). The adjustment variables indicated by DAG were as follows: sociodemographic characteristics (sex—male and female; age in years; level of education—less than 4 years, 4 years, or more), life habits (smoking habit—smoker, ex-smoker, and non-smoker; physical activity—yes or no), and food consumption (diet quality score). Those potentially mediators variables (functional disability and health conditions: number of diseases, self-rated health, hospitalization, and number of medications) were excluded from the analysis. In addition to these variables, an additional adjustment was made by BMI (calculated by dividing weight in kilograms by squared height in meters) in the model with WC to correct the effect of height.

Diet quality was assessed using the Healthy Eating Index (17) revised for the Brazilian population (IAS-R). The details are described in a previous publication (18).

### Data analysis

Descriptive analysis of the data was carried out through the distribution of absolute and relative frequencies and the estimation of measures of central tendency and dispersion. The normality of the distribution of the quantitative variables of interest was assessed using histogram, asymmetry and kurtosis coefficients, and the Kolmogorov–Smirnov test. In the bivariate analysis, the association of variables with death was assessed using Pearson’s chi-square test for the comparison of frequencies, Student’s t-test for the comparison of means, and the Mann–Whitney test for the comparison of medians.

Due to the inexistence of cutoff points for the anthropometric indices considered for older adults, a generalized additive model (GAM) was fitted to identify cutoff points for risk of death for WHtR and WC. GAMs are an extension of generalized linear models by including non-parametric smooth functions that loosen the relationship between a function of the mean of the dependent variable and the independent variables (19). This flexibility allows for the graphical evaluation of the curve shape and identification of cutoff points by analyzing inflection points. The GAM approach has been used for cutoff point definition in different studies (20–23).

The GAM with binomial distribution was used to evaluate cutoff points for the relationship between the anthropometric indicators of central adiposity and mortality. Smoothing parameters were selected by generalized cross-validation, using the mgcv package in R.

The variable time until the occurrence of death was defined as the time, in years, between the initial date (entry in the study) and the date of the event of interest (death) or end of the follow-up, that is, the date of the last contact (censorship). The starting date was considered to be the date of the first baseline interview. Participants whose deaths were not identified until the date of the last contact with the researchers (July 2018) were censored.

From the cutoff points obtained by GAM for the exposure variables, the independent association between adiposity and mortality was based on the hazard ratio estimates and its 95% confidence interval using Cox’s proportional hazards models. The assessment of the proportionality of risk over time was based on the Schoenfeld residuals plot, and, in the hypothesis test, the rejection of the null hypothesis (*p* > 0.05) indicates the proportionality of risk over time.

The significance level of *α* = 0.05 was used in all analyses. The analyses were performed with software Stata, v. 13.0 (Stata Corp., College Station, United States) and R 3.5.2.

## Results

During the follow-up period (9 years), 24.7% (197) of older adults died, 7.3% of these were due to CVD, and 7.5% (60) were not found. Of the deaths, 51.8% were men, with a mean age of 76.1 ± 9.0 years. Table 1 shows the baseline characteristics of older adults according to their survival. Compared to the survivors, the participants who did not survive were older (76.00 years old; interquartile range: 69.00–82.00), less educated (86.73%), and did not perform regular physical activities (81.63%). These differences were significant at a level of 5%.

**Table 1:** Characteristics of the older adults at baseline, according to survival. Viçosa, MG, Brazil, 2009–2018.

Characteristic	Total (*n* = 736)^†^	Survivor (*n* = 539)	Non-survivor (*n* = 197)	*p*-value*
Age in years, median (IIQ)	70.0 (64.0–77.0)	68.0 (64.0–74.0)	76.0 (69.0–82.0)	< 0.001
Sex, female, *n* (%)	393.0 (53.4)	298.0 (55.3)	95.0 (48.2)	0.09
Education less than 4 years, *n* (%)	595.0 (81.0)	425.0 (78.8)	170.0 (86.7)	0.02
Diet quality, average (SD)	64.5 (10.8)	64.8 (10.6)	63.8 (11.4)	0.30
Without physical activity, *n* (%)	522.0 (71.1)	362.0 (67.3)	160.0 (81.6)	<0.001
Current smoker, *n* (%)	74.0 (10.1)	51.0 (9.5)	23.0 (11.9)	0.09
Ex-smoker, *n* (%)	237.0 (32.3)	165.0 (30.6)	72.0 (37.1)	
BMI in Kg/m^2^, median (IIQ)	26.5 (23.7–29.6)	26.5 (23.9–29.6)	25.9 (23.0–29.1)	0.06
WC, median (IIQ)	95.2 (87.6–103.3)	95.1 (87.9–103.3)	95.4 (86.0–103.1)	0.71
WHtR, median (IIQ)	0.6 (0.5–0.6)	0.6 (0.5–0.6)	0.6 (0.5–0.6)	0.94

IIQ, interquartile range; SD, standard deviation; BMI, body mass index; WC, waist circumference; WHtR, waist-to-height ratio.

† The number may vary due to the presence of missing data. Education (*n* = 735); quality of the diet (*n* = 734); physical activity (*n* = 734); smoking (*n* = 733); BMI (*n* = 626); WC (*n* = 703); WHtR (*n* = 623).

* Pearson’s chi-square test for comparison between proportions, Student’s *t*-test for differences between means, and Mann -Whitney test for differences between medians.

**Figure 1:**
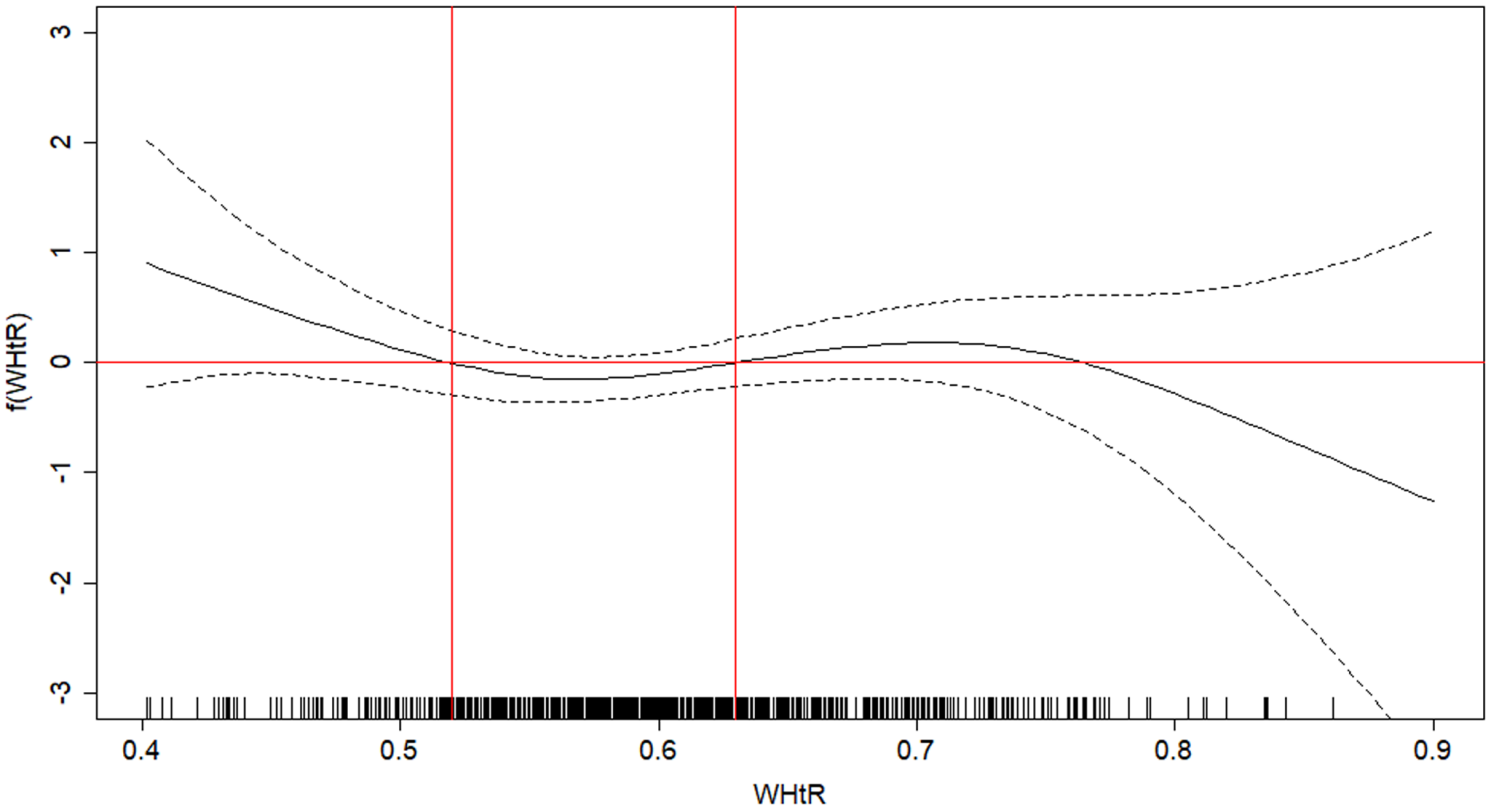
Attenuated function of mortality in older adults as a function of waist-to-height ratio (WHtR). Viçosa, MG, Brazil, 2009 -2018. The solid black line represents the regression line, and the dotted lines represent the 95% confidence interval. Cutoff points for WHtR = <0.52; between 0.52 and 0.63; ≥0.63.

**Figure 2:**
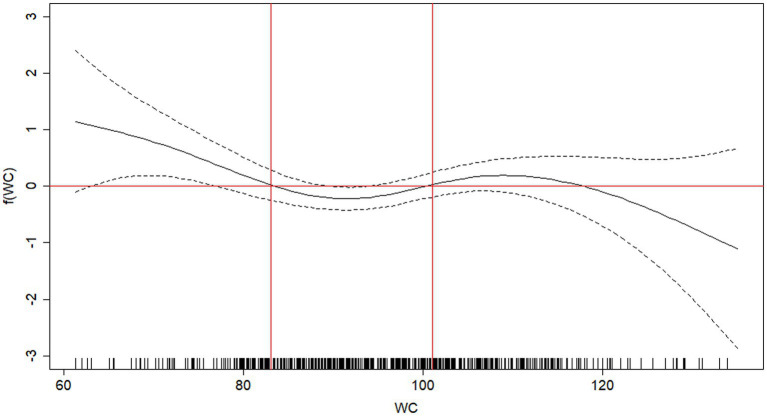
Attenuated function of mortality in older adults as a function of waist circumference (WC). Viçosa, MG, Brazil, 2009 -2018. The solid black line represents the regression line, and the dotted lines represent the 95% confidence interval. Cutoff points for WC: <83 cm; between 83 and 101 cm and ≥ 101 cm.

According to GAM analyses, the older adults with an increased risk of death were those with WHtR of <0.52 and ≥0.63 (Figure 1) and WC of <83 cm and ≥101 cm (Figure 2). When stratifying by sex, similar cutoff points for WC were found (Supplementary Figure 3).

The anthropometric indicators, WHtR and WC, were categorized according to the cutoff points estimated from the GAM, into normal (normal adiposity) and abnormal (high adiposity and very low adiposity).

Crude and adjusted hazard ratios, with the respective 95% confidence intervals for the relation between adiposity and all-cause mortality, are shown in Table 2. Older adults with WC of <83 cm had an increased risk of death only in the crude model (HR: 1.68, 95% CI: 1.12–2.52; *p* = 0.013). For the older adults with high WC (≥101 cm), significant associations were observed in model 2 after adjustment for sex, age, education, quality of diet, physical activity, and smoking (HR: 1.48, 95% CI: 1.05–2.09; *p* = 0.023), and when adjusted for variables of model 2 and BMI, an increase in mortality was indicated in those with greater adiposity (WC ≥ 101 cm—HR: 2.03, 95% CI: 1.20–3.41; *p* = 0.008). Older adults with high WHtR (≥0.63) had a higher risk of death when adjusted for the variables of model 2 (HR: 1.51, 95% CI: 1.01–2.26; *p* = 0.046). The assessment of the Schoenfeld residuals plot indicated proportionality of risk over time (*p* > 0.05).

**Table 2:** Simple and multiple analyses of the association between waist circumference (WC), waist-to-height ratio (WHtR), and mortality in older adults. Viçosa, MG, Brazil, 2009–2018.

Variable	Model 1^a^	Model 2^b^^,^^c^
	HR (CI 95%)	HR (CI 95%)
**WC (cm)** ^**†**^
≥83 e < 101	1	1
<83	1.68* (1.12–2.52)	1.51 (1.00–2.29)
≥101	1.29 (0.92–1.80)	1.48* (1.05–2.09)
**WHtR**
≥0.52 e < 0.63	1	1
<0.52	1.62 (1.00–2.64)	1.41 (0.86–2.33)
≥0.63	1.19 (0.82–1.74)	1.51* (1.01–2.26)

WC, waist circumference; WHtR, waist-to-height ratio; HR, hazard ratio; CI, confidence interval.

Sample size: 796; number of deaths: 197.

† Cutoff points for both sexes.

a Model 1: not adjusted.

b Model 2: adjusted for sex, age, education, quality of diet, physical activity, and smoking.

c Final model respects the proportionality of risks over time (WC: value of *p* = 0.96; WHtR: value of *p* = 0.45, Schoenfeld residuals).

* *p* < 0.05.

Crude and adjusted hazard ratios, with the respective 95% confidence intervals for the association between adiposity and CVD mortality, are shown in Table 3. In the unadjusted analysis, none of the indicators were significantly associated with CVD mortality (*p* > 0.05). For the older adults with high WC (≥101 cm), a significant association was observed in model 2 after adjustment for sex, age, education, quality of diet, physical activity, and smoking (HR: 2.09, 95% CI: 1.12–3.88; *p* = 0.02), and also when adjusted for the variables of model 2 and BMI, an increase in mortality was indicated in those with greater adiposity (WC ≥ 101 cm—HR: 4.99, 95% CI: 1.81–13.71; *p* = 0.002). The assessment of the Schoenfeld residuals plot indicated proportionality of risk over time (*p* > 0.05).

**Table 3:** Simple and multiple analyses of the association between waist circumference (WC) and waist-to-height ratio (WHtR) and CVD mortality older adults. Viçosa, MG, Brazil, 2009–2018.

Variable	Model 1^a^	Model 2^b^^,^^c^
	HR (CI 95%)	HR (CI 95%)
**WC (cm)** ^**†**^
≥83 e < 101	1	1
<83	1.52 (0.67–3.43)	1.39 (0.60–3.21)
≥101	1.74 (0.96–3.17)	2.09* (1.12–3.88)
**WHtR**
≥0.52 e < 0.63	1	1
<0.52	1.74 (0.68–4.45)	1.46 (0.56–3.80)
≥0.63	1.32 (0.64–2.76)	1.74 (0.79–3.85)

WC, waist circumference; WHtR, waist-to-height ratio; HR, hazard ratio; CI, confidence interval.

Sample size: 796; number of deaths: 197.

† Cutoff points for both sexes.

a Model 1: not adjusted.

b Model 2: adjusted for sex, age, education, quality of diet, physical activity, and smoking.

c Final model respects the proportionality of risks over time (WC: value of *p* = 0.82; WHtR: value of *p* = 0.75, Schoenfeld residuals).

* *p* < 0.05.

## Discussion

To the best of our knowledge, this study is the first to use generalized additive models to identify anthropometric cutoff points to predict the risk of death in Brazilian older adults. The results show that older adults with excess adiposity, WC (≥101 cm) and WHtR (≥0.63), have a higher risk of all-cause death and a higher risk of CVD death. This risk was observed for higher values of these indicators than those usually adopted in adults.

Aging is associated with a process that leads to the redistribution of fat with an increase in abdominal fat (24), predisposing the individual to risk factors for the development of cardiometabolic diseases (25). WC has been frequently used to assess abdominal adiposity for being a simple alternative to capture information on abdominal fat distribution and may be less affected by variation in lean mass (26). However, WC does not consider height; thus, shorter individuals with a determined WC may have a higher cardiometabolic risk than taller individuals with the same WC (27). Despite this limitation, studies show that increased WC is positively associated with mortality in adults and older adults (12, 28).

Adjustment for BMI may decrease confounding by pre-existing diseases that are associated with losing weight due to lean rather than fat mass loss (26, 29). Cehan et al. (26) observed that the analysis of the joint effect of waist circumference and BMI on mortality supports the linear association for waist circumference after accounting for BMI.

In the present study, an increased risk of death was observed in older adults with increased WC when the model was adjusted by BMI, that is, the adjustment for BMI strengthened the association of waist circumference with mortality, which was previously reported (4, 26, 28). Beleigoli et al. (4) investigated the relationship between adiposity and mortality in Brazilian older adults over a period of 10 years and found a non-significant association between WC and mortality; however, after adjusting the WC by BMI, the association became marginally positive. A similar result was found in another study with American adults and older adults. The authors found that WC was positively associated with higher mortality when adjusted by BMI, highlighting that this anthropometric measure must be evaluated in combination with BMI to assess the risk of death related to adiposity (26). A cohort study with Brazilian older adults (13) and a meta-analysis of older adult cohorts also found that high WC values were associated with a higher risk of CVD mortality, which is similar to after adjusting for BMI (28).

Because body composition changes with aging, WHtR can be an alternative index, as it is valid for diagnosing obesity in older adults (30) and accurate in the discrimination of visceral adiposity (31). The use of this index is scarce in studies on mortality. However, some studies indicate a relationship between central adiposity and higher mortality in the older population (12, 32, 33). Our results corroborate these findings.

In the present study, there was an absence of a linear relation between anthropometric indicators and mortality from all causes, as well as the non-significant difference in the cutoff points for WC by sex, which reinforces the need to define specific cutoff points for older adults. Therefore, the cutoff points identified for adiposity in risk of death in this study were WC ≥ 101 cm and WHtR ≥0.63. This finding contrasts with literature that defines visceral adiposity and cardiometabolic risk for adults as WC ≥ 94 cm and ≥ 80 cm for men and women, respectively (34), and cardiometabolic risk for WHtR values above 0.50 in both sexes, for adults (35). However, due to the sample size, stratified analyzes by sex were not performed, considering that the study was not primarily designed to answer this question. Otherwise, we performed the GAM analysis stratified by sex, and the cutoff points are very similar (Supplementary Figure 3). Another point to be discussed is the lack of cutoff points for lower limits. Considering the peculiarities of older individuals, low adiposity can determine the risk of death. In a previous study with the same sample, we found that underweight and low muscle reserves were significantly associated with higher mortality. We observe that cutoff points for calf circumference of <34.5 cm and BMI of <18.5 kg/m^2^ were significantly associated with a higher risk of death (23). It is possible that the sample size of the present study did not have enough power to demonstrate this association. Subsequent studies with adequate sample sizes may be useful to improve the evidence on this issue.

Considering the absence of cutoff points for these indicators in older adults and the identification, by GAM, of higher values compared to those for adults, there is a reduction in false positives, that is, older adults who would be identified with a higher risk of death due to adiposity do not have this increased risk. This fact suggests that, to some extent, adiposity does not represent a risk of death for these individuals.

It is important to consider that the estimated cutoffs for anthropometric measures are valid for this population group, and it is not adequate to generalize. Subsequent studies with other older populations could be performed to validate specific cutoff points.

In this study, we chose not to use BMI because of its limitations as a measure of adiposity in older adults, which may explain the reduced risk of death in the participants with excess weight since it does not reflect changes due to aging in body composition such as loss of muscle mass and increase of fat, mainly visceral, as well as the decline in height (14). Thus, indexes such as WC and WHtR are more adequate to identify adiposity than BMI, as they have a strong correlation with intra-abdominal fat and cardiometabolic risk factors (36, 37). In this sense, in order to elucidate the relation between adiposity and mortality, it was observed that WC and WHtR are good predictors of mortality in the older population.

The main limitation of this study is the absence of repeated measurements of anthropometric indices, which makes it impossible to analyze possible changes over time. Another limitation is the lack of more precise techniques, such as computed tomography, magnetic resonance imaging, ultrasound, and dual-energy X-ray absorptiometry (DXA), to assess adiposity. However, since the equipment used for a more accurate adiposity assessment is not available in the daily healthcare of older adults, it can be reasonably estimated with easily obtainable measures. It is important to consider that the sample size was not calculated to estimate mortality. Thus, the results must be interpreted cautiously since it was not possible to estimate associations between some exposures (low adiposity) and mortality, as well as not being possible to stratify by sex. In addition to that, the results cannot be extrapolated to all Brazilian older adults.

Finally, this study showed that excess adiposity, assessed by WHtR and WC, considering specific cutoff points for older adults, was associated with an increased risk of death, including mortality by cardiovascular diseases. These results point to the relevance of anthropometric measures in the evaluation and monitoring of health in aging.

## Data availability statement

The raw data supporting the conclusions of this article will be made available by the authors, without undue reservation.

## Ethics statement

The studies involving human participants were reviewed and approved by Human Research Ethics Committee of the Federal University of Viçosa/MG, Approval Reference Number: 65782817.1.0000.5153. The patients/participants provided their written informed consent to participate in this study.

## Author contributions

DF worked in study design, data collection, analysis and interpretation of data, article writing, and critical review of relevant intellectual content. AR supervised the design, collection and analysis and interpretation of data, collaborated in the article writing, and critical review of relevant intellectual content. LL collaborated in the analysis and interpretation of data, article writing, and critical review of relevant intellectual content. AN collaborated in the analysis and interpretation of data and relevant critical review of the intellectual content. ÂS collaborated in the data collection and critical review of relevant intellectual content. All authors contributed to the article and approved the submitted version.

## Funding

The present study was supported by the Fundação de Amparo à Pesquisa do Estado de Minas Gerais (FAPEMIG) (Process APQ-00594-17). The funder had no role in the design, analysis, or writing of this article.

## Conflict of interest

The authors declare that the research was conducted in the absence of any commercial or financial relationships that could be construed as a potential conflict of interest.

## Publisher’s note

All claims expressed in this article are solely those of the authors and do not necessarily represent those of their affiliated organizations, or those of the publisher, the editors and the reviewers. Any product that may be evaluated in this article, or claim that may be made by its manufacturer, is not guaranteed or endorsed by the publisher.

## Footnotes

^1^ http://www.dagitty.net/
