# Supplementary material for: Central adiposity as a predictor of mortality in older adults: Identification of cutoffs using generalized additive models
Source: Front Nutr. 2023 Apr 18;10:1132006. doi: 10.3389/fnut.2023.1132006 (PMC10151932; doi:10.3389/fnut.2023.1132006)
Supplement: Supplementary file 1 [file Data_Sheet_1.pdf]

Supplementary Material

Central adiposity as predictor of mortality in older adults: identification of cutoffs using generalized additive models

Dalila Pinto de Souza Fernandes*, Leidjaira Lopes Juvanhol, Aline Araújo Nobre, Ângela Maria Natal de Souza, Andréia Queiroz Ribeiro.

*** Correspondence:** Corresponding Author: dalilaf.ufv@gmail.com

1 Supplementary Data

Supplementary Table 1: Comparison by sex and age between participants and non-participants in the study. Viçosa, MG, Brazil, 2009-2018.

Variable	Participants		Total	p value*
	Yes (n=796)	No (n= 62)	(n=858)	
**Sex, n (%)**				
Female	421 (93.3)	30 (6,7)	451 (100.0)	0.512
Male	375 (92.1)	32 (7,9)	407 (100.0)	
**Age (years), n (%)**				
60-69	390 (95.4)	19 (4.6)	409 (100.0)	
70-79	282 (92.2)	24 (7.8)	306 (100.0)	0.002
≥ 80	124 (86.7)	19 (13.3)	143 (100.0)	

*Pearson's chi-square test.

There are no significant differences between participants and non-participants in terms of sex. On the other hand, non-participants are, on average, older than participants.

2 Supplementary Figures

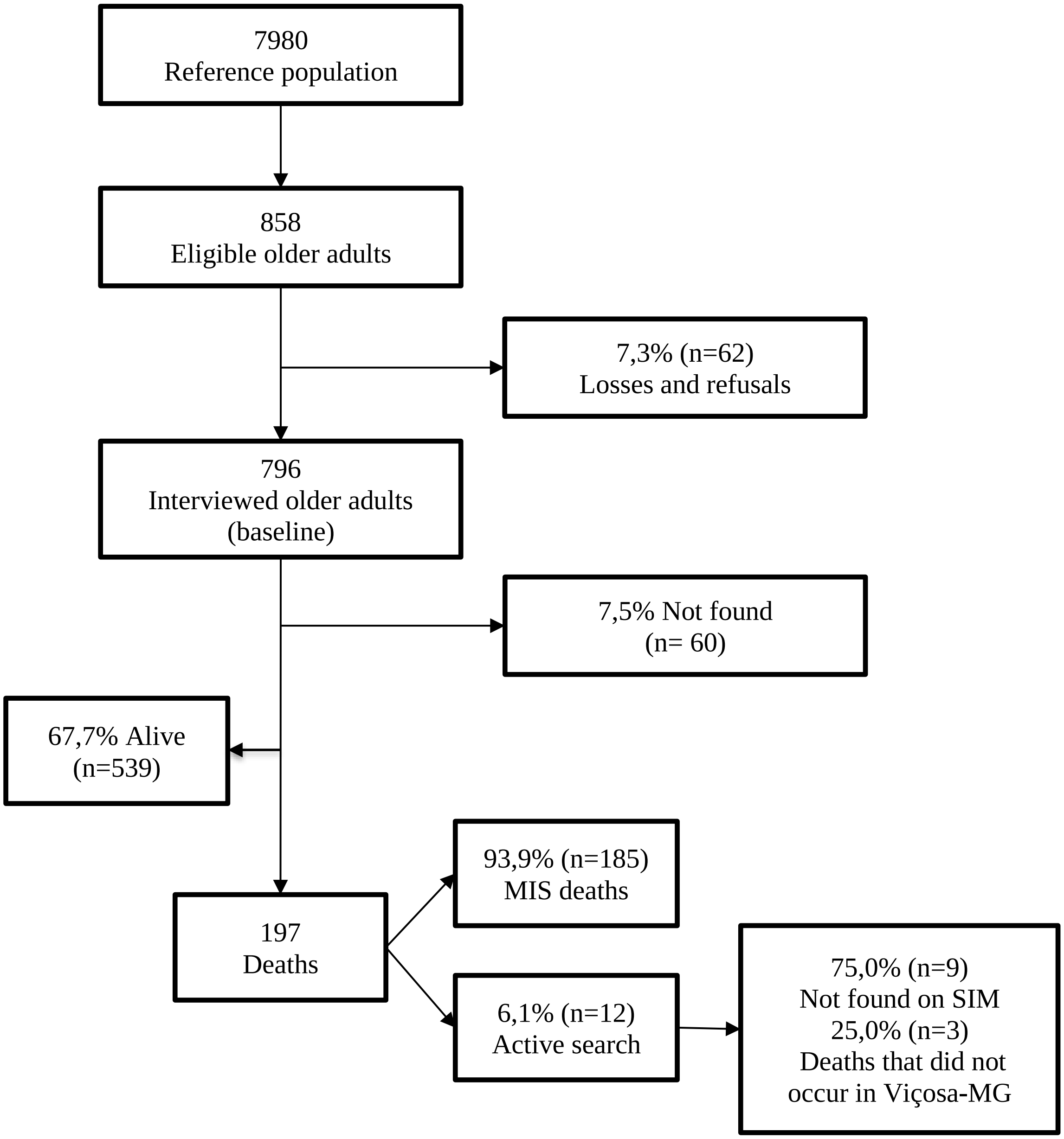

Supplementary Figure 1: Sample selection flowchart for the study with the elderly (n= 796). Viçosa, MG, Brazil, 2009-2018.

Note:

SIM: Brazilian Mortality Information System

In the theoretical model represented by a directed acyclic graph (DAG), the exposure and outcome variables are represented by the symbols ► and |, respectively. The pink circles indicate variables that precede both exposure and outcome; the blue circles represent the variables that precede only the outcome. The green lines denote causal paths and the pink lines characterize the paths that potentially skew the estimates of the studied association (confounders).

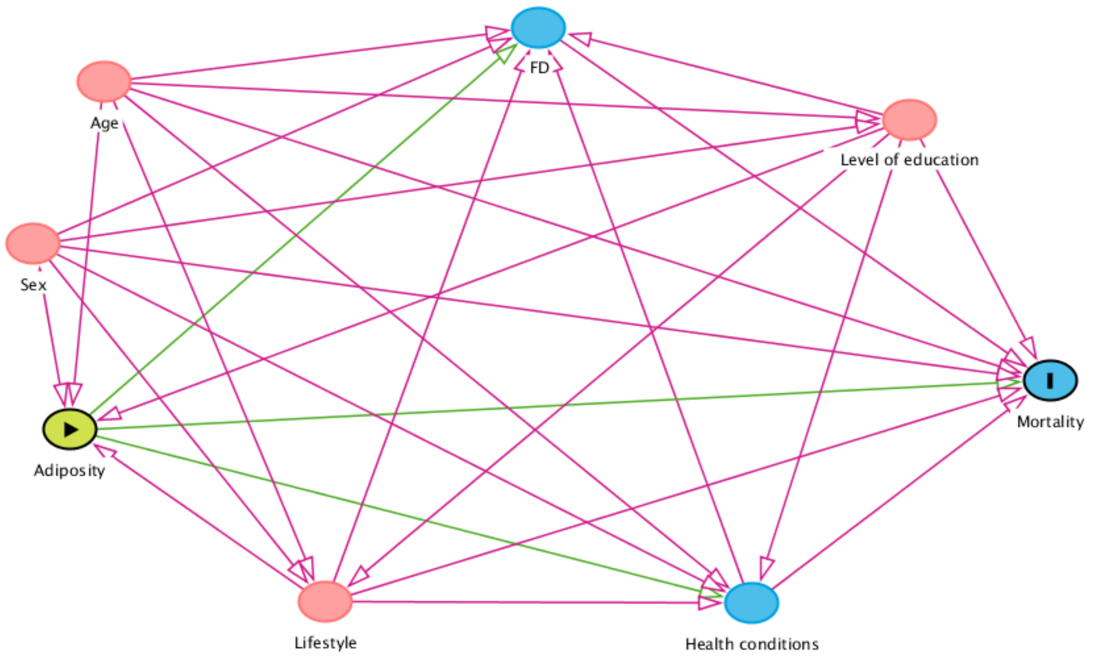

Supplementary Figure 2. Acyclic graph directed (DAG) to assess the association between adiposity and mortality. Viçosa, MG, Brazil, 2009–2018.

Note:
FD (functional disability).
Sociodemographic: sex, age and level of education.
Health conditions: self-reported diseases, self-rated health, hospitalization, number of medications.
Lifestyle: smoking habit, physical activity and food consumption.

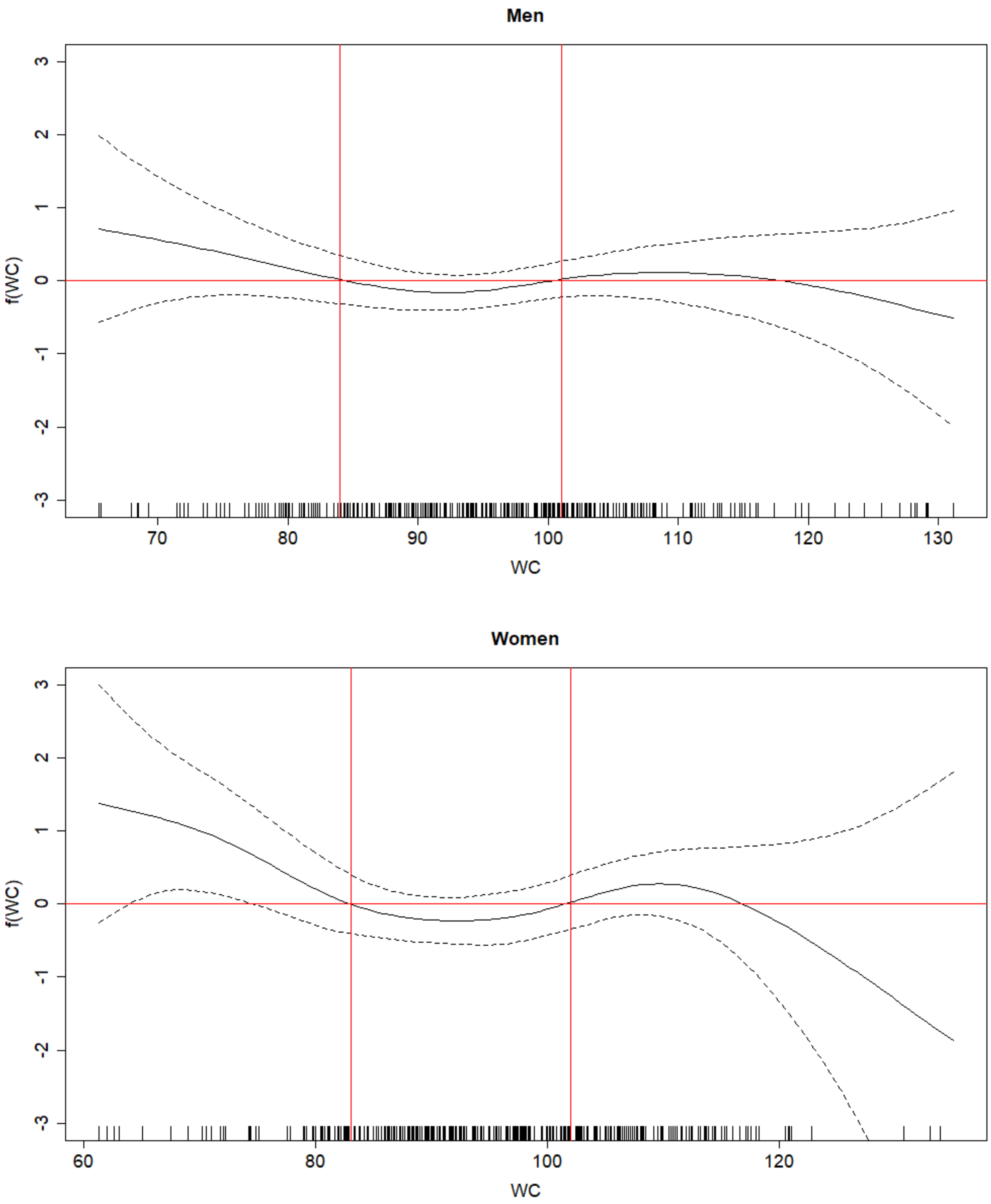

Supplementary Figure 3. Attenuated function of mortality in the older adults according to waist circumference by sex. Viçosa, MG, Brazil, 2009–2018.

Note:

The solid black line represents the regression line and the dotted lines represent the 95% confidence interval.

Cutoff points for WC in men: <84 cm; between 84 and 101 cm; ≥ 101 cm.

Cutoff points for WC in women: <83 cm; between 83 and 102 cm; ≥ 102 cm.
